# Supplementary material for: Development Control and Inactivation of Byssochlamys nivea Ascospores by Hyperbaric Storage at Room Temperature
Source: Foods. 2023 Feb 25;12(5):978. doi: 10.3390/foods12050978 (PMC10001197; doi:10.3390/foods12050978)
Supplement: Supplementary file 1 [file foods-12-00978-s001.zip › foods-2229813-SI.pdf]

Supplementary Materials

Table S1. Impact of different pre-activation steps in inactivation kinetics of *B. nivea* ascospores

Activation step	Pressure (MPa)	D-Value (Days)	R^2	RMSE	Z-value (MPa)	R^2	RMSE	Note***
No activation	50	26.43 ± 6.01	0.7762	0.2234	-	-	-	-
	75	22.41 ± 2.46	0.9407	0.1240				
Thermal activation (80 ºC/30 sec)	25	29.92 ± 4.47	0.8222	0.1789	91.69 ± 38.75	0.8480	0.4102	D-value obtained from the 2nd day of storage onwards
	75	2.50 ± 0.71	0.8609	0.7952				D-value obtained from the 1st to the 10th day of storage
	150	1.11 ± 0.04	0.9987	0.0941				D-value obtained from the 1st to the 5th day of storage
HPP activation (600 MPa, 3 min)	25	2.79 ± 0.17	0.9928	0.0810	110.43 ± 37.66**	0.8958	0.1092	D-value obtained until the 5th day of storage
	50*	1.22 ± 0.33	0.9332	0.3103				D-value obtained until the 2nd day of storage
	50*	21.82 ± 1.43	0.9957	0.0172				D-value obtained from the 2nd to the 10th day of storage
	75	0.99 ± 0.08	0.9932	0.1184				D-value obtained until the 2nd day of storage
	150	1.03 ± 0.56	0.7693	0.7512				D-value obtained until the 2nd day of storage

*Biphasic behaviour

**Z-value obtained considering only the first D-value of 50 MPa, and excluding the D-value from 150 MPa

***D-values were obtained using a minimum of three experimental points

Figure S1

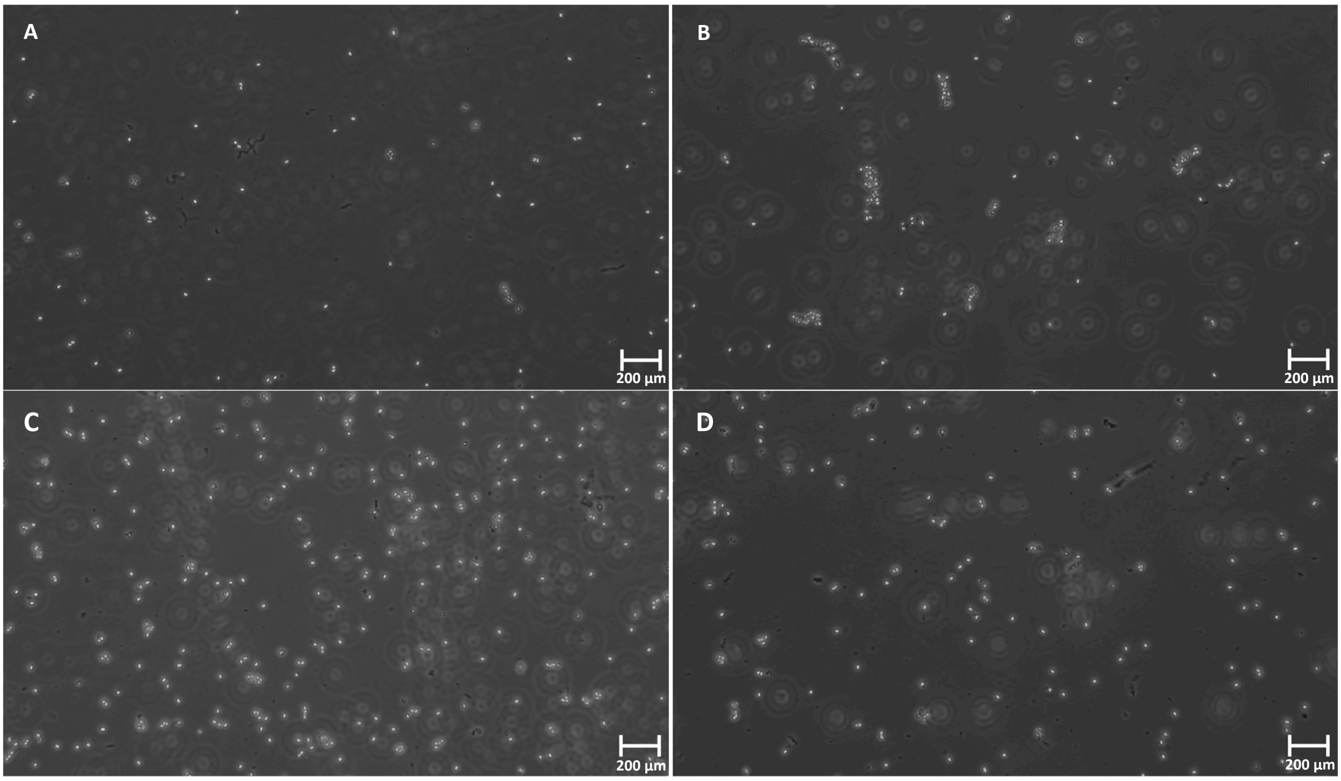

Figure S1. Phase contrast microscopy images (20X magnification) of the commercial apple juice inoculated with *B. nivea* ascospores in unpasteurized samples (A), thermally pasteurized samples at 70 °C/30 sec (B) and 80 °C/30 sec (C), and HPP- pasteurized samples at 600 MPa/3 min, 17 °C (D) at the beginning of the storage experiments.
